# When Do Antibiotics Induce “Resistance”?

**DOI:** 10.1155/S1064744996000221

**Published:** 1996

**Authors:** Gilles R. G. Monif

**Affiliations:** Department of Obstetrics and Gynecology Creighton University School of Medicine 601 North 30th Street Suite 4700 Omaha NE 68131 USA


Infectious Diseases in Obstetrics and Gynecology 4:102–103 (1996)
© 1996 Wiley-Liss, Inc.

### Letter to the Editor: When Do Antibiotics Induce "Resistance"?

To the Editor:

McDuffie et al.[1] have reported 4 cases of Enterobacteriaceae chorioamnionitis occurring in gravidas who received ampicillin prophylaxis for premature rupture of the fetal membranes and group-B streptococcus carriage. Three of the isolates were ampicillin-resistant *Escherichia coli.* The fourth case was due to *Klebsiella pneumoniae.* Two of the resultant neonates died with fulminant perinatal septicemia. The rationale for the publication of their manuscript was the contention that these were examples of "adverse perinatal outcomes due to selection or overgrowth of resistant organisms resulting from the use of ampicillin." More recently, Amstey and Gibbs[2] have written an editorial opinion in which they have contended that the "favorable pharmacokinetics of penicillin G and its narrow and specific spectrum results in diminished potential for selecting more resistant organisms (in comparison to ampicillin)."

### INTRINSIC VS. INDUCED RESISTANCE

### Induced Resistance

The prolonged use (especially associated with suboptimal dosing) of an antibiotic can allow the emergence of a resistant subpopulation of a previously susceptible strain which then becomes the dominant population. An example of this phenomenon is the abuse of kanamycin during the 1970s in newborn intensive care units (NICUs), resulting in *K. pneumoniae* isolates that were resistant to virtually all the aminoglycosides available at that time. Another example of induced resistance is the change in the avidity of the binding of penicillin to the penicillin-binding proteins of selected strains of *Streptococcus pneumoniae.*

If induced resistance to ampicillin had occurred within the discipline of obstetrics and gynecology, its emergence would have been documented in NICUs. Virtually every infant suspected of sepsis receives ampicillin and gentamycin. The medical and nursing staff in these units would have become vectors for resistant gram-positive bacteria whose ultimate summation would have been epidemics of bacteria that were traditionally susceptible to ampicillin but subsequently resistant.

### Intrinsic Resistance

If an antibiotic is used against a bacterium whose spectrum of susceptibility is not encompassed by that antibiotic, one cannot anticipate having a true biologic effect. Approximately 35–40% of all current *E. coli* isolates are resistant to ampicillin. This resistance is mediated primarily by the presence of significant quantities of β-lactamases within the periplasmic space. Over 95% of all *K. pneumoniae* isolates are similarly inherently resistant to ampicillin. When isolated instances of disease due to Enterobacteriaceae occur in the face of ampicillin therapy, the probability is that the same pattern of disease would have been observed whether ampicillin had been given or not. Unless one is dealing with a phenomenon such as anaerobic progression or the use of antibiotics which have an impact on 30 or 50S ribosomes, noneffective antibiotics will not alter the progress of monoetiologic disease.

Chorioamnionitis is a community-acquired infection. The bacteria causing this disease are almost invariably brought into the hospital by the patient per se. The susceptibility of a given isolate reflects the susceptibility of the genus in the community. In an unpublished review (Monif, unpublished data) of chorioamnionitis and perinatal septicemia dating from the early 1970s (when the therapy was penicillin and kanamycin or ampicillin), ampicillin was ineffective in only 4 of 61 cases. In each of these 4 cases, the bacterial resistance to ampicillin was noninduced. The isolates were 1) β-lactamase-producing *Haemophilus influenzae,* 2) *K. pneumoniae,* 3) resistant *E. coli,* and 4) *Enterobacter cloacae.*

---

Address correspondence/reprint requests to Dr. Gilles R.G. Monif, Department of Obstetrics and Gynecology, Creighton University School of Medicine, 601 North 30th Street, Suite 4700, Omaha, NE 68131.

Received July 9, 1996
Accepted July 10, 1996

Drug chemoprophylaxis with ampicillin alters the incidence of disease by diminishing the denominator, thus magnifying the impact of the numerator. As a consequence, the perception of the relative importance of an isolate is changed. In the setting described by McDuffie et al.,[1] ampicillin did not select a resistant organism to produce disease, but merely altered the proportionality of resistant to nonresistant isolates within the equation. McDuffie et al.[1] identified 4 cases in the numerator without regard to impact of drugs on the denominator. The report failed to identify the time frame over which these cases were collected, the projected number of cases of chorioamnionitis or perinatal septicemia which would have been successfully treated or aborted by ampicillin, or the presence or absence of asymptomatic bacteriemia.

### So What?

Why is there the need to clarify conceptual thought? Once an idea is published without challenge, it has potential to take on a life of its own. In 1994 and 1996, the Centers of Disease Control[3,4] published in the *Federal Register* their preliminary proposal for addressing the eradication or reduction of early-onset group-B streptococcal neonatal disease. In the Executive Summary of the committee's recommendations for limiting the use of intrapartum chemoprophylaxis is the following statement (citing McDuffie et al.[3]): "... limit the use of antimicrobials to about 5% of all deliveries, thus minimizing maternal side effects and the emergence of antimicrobial-resistant organisms."

Good intentions may become dangerous due to conceptual errors.

*Gilles R.G. Monif*
*Department of Obstetrics and Gynecology*
*Creighton University School of Medicine*
*Omaha, Nebraska*

### REFERENCES

1. McDuffie RS, McGregor JA, Gibbs RS: Adverse perinatal outcome and resistant Enterobacteriaceae after antibiotic usage for premature rupture of the membranes and group B streptococcus carriage. *Obstet Gynecol* 82:487–489, 1993.
2. Amstey MS, Gibbs RS: Is penicillin G a better choice than ampicillin for prophylaxis of neonatal group B streptococcal infections? *Obstet Gynecol* 84:1058–1059, 1994.
3. Centers for Disease Control and Prevention: Prevention of group B streptococcal diseases: A public health perspective. *Fed Regist* 59:64764–64773, 1994.
4. Centers for Disease Control and Prevention: Prevention of perinatal group B streptococcal disease: A public health perspective. MMWR 45(RR-7):1–24, 1996.

